# The effect on potential adverse drug events of a pharmacist-acquired medication history in an emergency department: a multicentre, double-blind, randomised, controlled, parallel-group study

**DOI:** 10.1186/s12913-015-0990-1

**Published:** 2015-08-20

**Authors:** Jesus Becerra-Camargo, Fernando Martínez-Martínez, Emilio García-Jiménez

**Affiliations:** Universidad Nacional de Colombia, Ciudad Universitaria Edificio 450 Oficina 204, Bogota, 111321 Cundinamarca Colombia; Pharmacy Department, Universidad de Granada, Granada University’s Pharmaceutical Care Research Institute, Paseo Cartuja, S/N, 18071 Granada, Spain

## Abstract

**Background:**

Potential adverse drug events (PADEs) are defined as being potentially harmful unintentional medication discrepancies. Discrepancies regarding medication history (MH) often occur when a patient is being admitted to a hospital’s emergency department (ED); they are clinically important and represent a significant source of data regarding adverse drug events occurring during emergency admission to hospital. This study sought to measure the impact of pharmacist-acquired MH during admission to an ED; it focused on whether a patient’s current home medication regimen being available for a doctor when consulting a patient in an ED would have reduced potential adverse drug events.

**Method:**

A multicentre, double-blind, randomised, controlled parallel-group study was carried out at 3 large teaching hospitals in Bogota, Colombia. Two hundred and seventy patients who had been admitted to an ED were enrolled; each had a standardised, comprehensive MH interview, focusing on a patient’s current home medication regimen prior to being seen by a doctor. Data recorded on the admission medication order form was available to be used by a doctor during consultation in the ED. The main outcome dealt with comparing the intervention and control groups regarding the percentage of patients having at least 1 potential adverse drug event.

**Results:**

There were 811 PADE (3.35 per patient), 528 (65 %) on the standard care arm and 283 (35 %) on an intervention arm. Most PADEs were judged to have had the potential to cause moderate discomfort (42.6 %), 33.4 % were deemed unlikely to have caused harm and 23.9 % were judged to have had the potential to cause clinical deterioration.

**Conclusion:**

Many patients suffer potentially adverse drugs events during the transition of care from home to a hospital. Patient safety-focused medication reconciliation during admission to an ED involving a pharmacist and drawing up a history of complete medication could contribute towards reducing the risk of PADES occurring and improve follow-up of patients’ medication-based therapy.

**Trial registration:**

28/10/2012, ISRCTN63455839.

## Background

Potential adverse drug events (PADE) are defined as being potentially harmful, unintentional medication discrepancies [1]. It has been estimated that such events account for 17 million emergency department (ED) visits and 8.7 million hospital admissions annually in the United States [2, 3]. They are clinically important and represent a significant source of PADE occurring during emergency admission to hospital. Adverse drug-related events have recently been evaluated in ED care settings; it has been estimated that 12 %–14.2 % of hospital admissions are drug-related [4]. At least 1 medication is omitted in more than 57 % of patients admitted to an ED [5, 6]. Involving a pharmacist-obtained MH has been associated with a 43 % to 84 % relative risk reduction [7–10].

Unfortunately, the effect on PADEs on a pharmacist-acquired medication history in an ED has not reflected in most studies as these have been retrospective or have analysed administrative data. Retrospective studies may underestimate the incidence of drug-related visits because information may be missing or has been inaccurately documented because patients seen in the ED for an adverse drug-related event are typically not admitted [11]. Studies performed to date have used different concepts regarding PADE, thereby limiting comparative evaluation and generalizability [12].

Despite the burden of drug-related morbidity and mortality, prospective research assessing the potential clinical importance of such discrepancies and/or the impact on PADEs of an MH acquired by a pharmacist in an ED has been limited. An attempt was made to overcome some research limitations in this area by using a prospective design aimed at determining whether PADE could become reduced by a pharmacist-acquired MH in an ED which focused on a patient’s current home medication regimen and which was available for a doctor when consulting a patient in an ED.

## Methods

### Study design, setting and participants

**Fig. 1:**
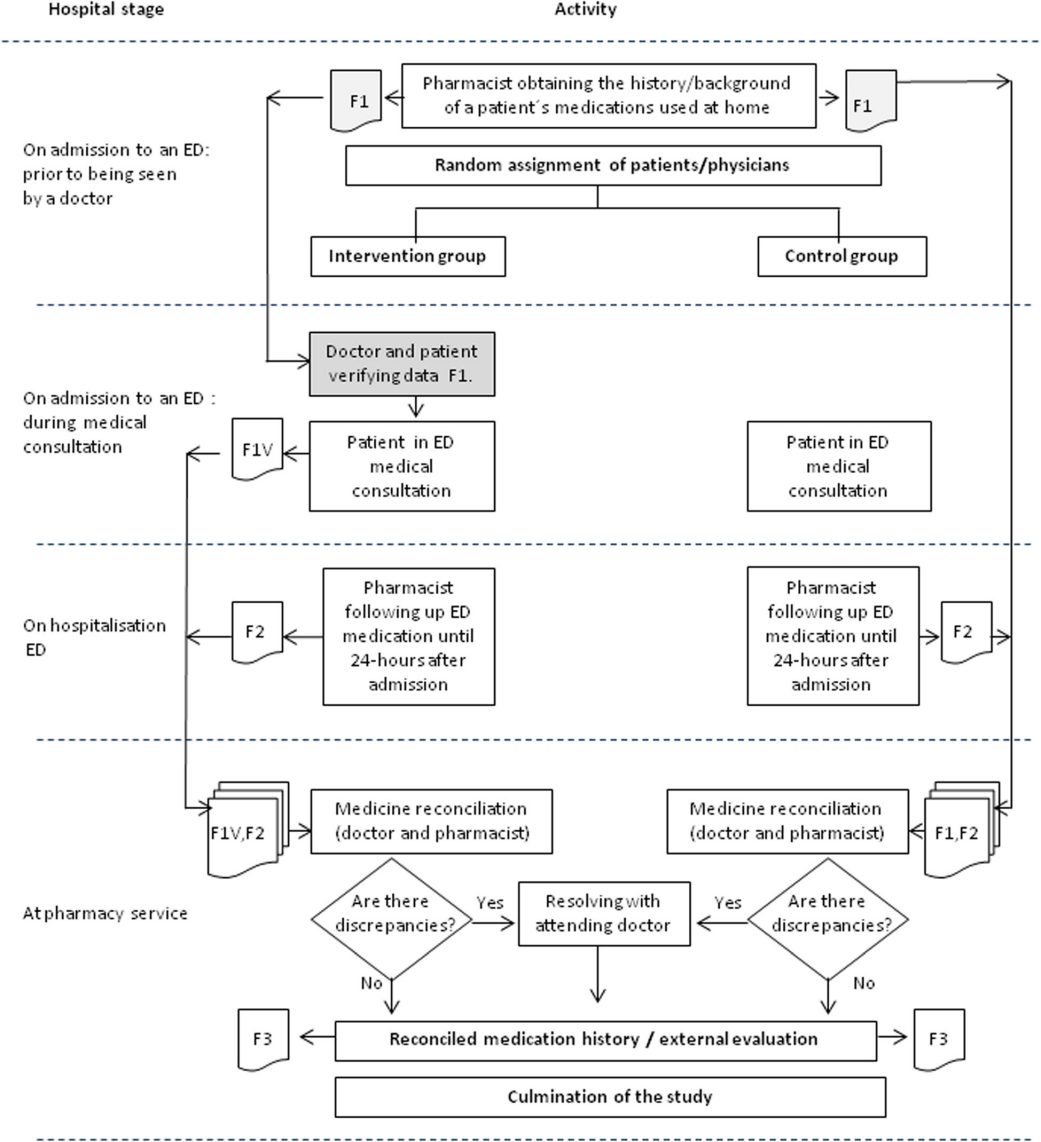
Study design

A multicentre, double-blind, randomised, controlled parallel-group trial study was carried out from October 26^th^ to November 30^th^ 2012 at 3 large teaching hospitals in Bogota, Colombia; Fundacion Cardio Infantil, San Carlos teaching hospital and Samaritana teaching hospital. Each participant gave their written informed consent and the study protocol was approved by the hospitals’ ethics committees. A full description of the study design has been published previously [6]. All consecutive patients (18 years or older) who had been admitted to an ED, were taking at least one medication or who had been prescribed a minimum of one prescription medication before admission and who had been hospitalised for at least 24 h were eligible for inclusion in this study. Patients were randomly assigned to an intervention or standard care arm using computer-generated random numbers (Microsoft Excel). Doctors who received patients were also randomly allocated; each randomisation manager made a daily allocation which depended on the number of doctors and residents per shift. A nurse (epidemiologist) at each site who was not involved in caring for the trial patients and independent of the site investigator was responsible for trial allocation and record-keeping (i.e. the randomisation manager). (Fig. 1)

### Ethics approval

The protocol and supporting documents were reviewed, approved and registered by the following Ethics Committees for Clinical Research: Fundacion Cardio Infantil (DDI-376, September 18^th^, 2012), San Carlos teaching hospital (FHS C-OCC 100–12, August 13^th^ 2012), and the Samaritana teaching hospital (142, June 27^th^, 2012).

### Intervention

The intervention consisted of a pharmacist acquiring patients’ medication histories in an ED prior to their being seen by a doctor. It focused on a patient’s current home medication regimen which was documented on an admission medication order form which was available for use by a doctor when consulting a patient in an ED. The admitting doctors verified the data with patients and indicated which home medications were to be reordered, suspended or discontinued.

### On admission to an ED

A pharmacist held a standardised, comprehensive MH interview during ED admission, focusing on the current home medication regimen for all the patients included in the study, prior to being seen by a doctor. A thorough history of all regular medication use was ascertained, using all the following sources of information: patient and/or caregiver interview, a check of the last prescription and an inspection of the medicines carried by a patient (i.e. in the ED). Pharmacists conducted telephone interviews with caregivers or family members when patients were unable to clarify their medication regimen. This data was recorded on the admission medication order form.

The medication order form was then used by a doctor during consultation for issuing prescriptions in an in-patient ED (just for the intervention group). The doctor checked boxes to verify data with a patient and indicated which home medications were to be reordered, suspended or discontinued. This resulted in an accurate and comprehensive history of patients’ current home medication regimens. Relevant demographic and medical data was collected and documented.

Another pharmacists blinded to intervention status reviewed each medical chart regarding all the drugs prescribed 24 h after having been admitted to an ED. The data came from various information sources including a patient’s computerised hospital medical record, the admission medication orders, the physician-recorded MH, the nurse-recorded MH, interviews with patients, medication administration records and demographic information. The pharmacist also attempted to verify with patients if any medication changes had been made since their clinical assessment (i.e. on admission to an ED). This was documented in the list of medications prescribed by a doctor during 24 h in an ED (F2).

### Standard group

Control group patients received standard care; this included doctors documenting medication histories in admission notes and nurses reviewing medication orders for appropriateness. The admission medication order form was given to the doctors at a later stage for them to amend prescriptions made on admission. Pharmacists would not have been routinely involved in documenting patients’ medication histories on admission to the institutions involved in the present study; this function is primarily the admitting resident doctor or a medical student’s responsibility.

### Medicine reconciliation process

A patient’s current home medications were compared to medications prescribed 24 h after having been admitted to an ED to see whether a patient’s home medications had also been prescribed by a doctor in an ED. This was done by an independent team consisting of a pharmacist and a doctor blinded to intervention status. The whole team received formal MedRec training, including a description of data-collection tools and procedures. External evaluation was made by the chief of each hospital’s ED after MedRec had ended; this person then resolved any discrepancies with each doctor. If incongruity was detected and the reason had not been documented in the medical record, this was clarified with the medical team and the patients so involved. If needed, a pharmacist contacted a particular patient or ED doctor to clarify any unclear medication regimen. Following MedRec, medication continuation required that doctors write a separate medication order.

The MedRec history (F3) (i.e. a gold standard) thus provided an accurate and up-to-date MH for avoiding discrepancies, such as omissions, duplications, dose errors or drug interactions. This ensured that the medication list received by the next ward was correct.

### Outcome

The intervention dealt with comparing the percentage of patients in the intervention and control groups having at least 1 PADE. A secondary outcome was recording the number of PADEs per patient using Poisson regression analysis.

### Preparing PADE summaries

Discrepancies between a patient’s home medication and admission ED orders were identified and intentional reasons for making changes were sought from the medical record. Clearly unintentional medication discrepancies were recorded. The doctor blinded to intervention status and a pharmacist involved in MedRec prepared a table giving a detailed description of medication discrepancies, including prescribed medication, drug class and type of discrepancy which could have been associated with any of the following: drug, dosage, frequency, administration route, appropriateness of restarting medication, therapeutic duplicity and/or medications lacking indication for use. The list was independently sent to two reviewers who judged each medication discrepancy for its potential to cause harm.

### Determining the potential to cause harm

Such medication discrepancies’ clinical severity was independently assessed by two clinical pharmacists blinded to the patient data collection forms. Classifying the degree of effect was adapted from the method used by Cornish *et al*. [13]. A Class 1 discrepancy was unlikely to result in clinical deterioration. An example would be a patient being prescribed 10 mg/d of desloratadine on admission, despite a 5 mg/d dosage having been reported during the interview. Class 2 discrepancies were those having the potential to cause moderate clinical deterioration. An example would be a patient for whom 10/d mg atorvastatin and 20 mg/d omeprazole had been omitted from the drugs prescribed on admission, despite such patient having reported that these were frequently taken at home during the interview. Class 3 discrepancies would have resulted in a patient’s severe clinical deterioration. An example would be when a cardiac arrhythmia patient had been admitted to hospital and been prescribed 150 mg/d propafenone despite having reported that he had been taking propafenone during the interview; however, the ED doctor did not know that a lower dose (half of that prescribed) had already been ordered by a cardiologist 3 weeks earlier. If agreement was not found, an internist independently rated the event and consensus was reached regarding all discrepancies.

### Statistical methods

Fleiss’ kappa coefficient was used for assessing the level of agreement among evaluators when judging PADEs. Patients’ characteristics were calculated using percentages, means, standard deviations and inter-quartile ranges. The number of PADEs per patient was identified by an exact *X*^2^ test to investigate differences between treatment groups regarding the percentage of patients having at least 1 PADE. Univariate and multivariate logistic regression analysis was used to investigate predictors of at least 1 PADE and analyse the risk ratio between the intervention and control groups. Poisson regression analysis was used to determine associations between the number of PADEs per patient and study group characteristics. All tests were 2-tailed and a p < 0.05 test result was deemed statistically significant. All statistical analysis involved using R statistics software.

## Results

### Participant flow

**Fig. 2:**
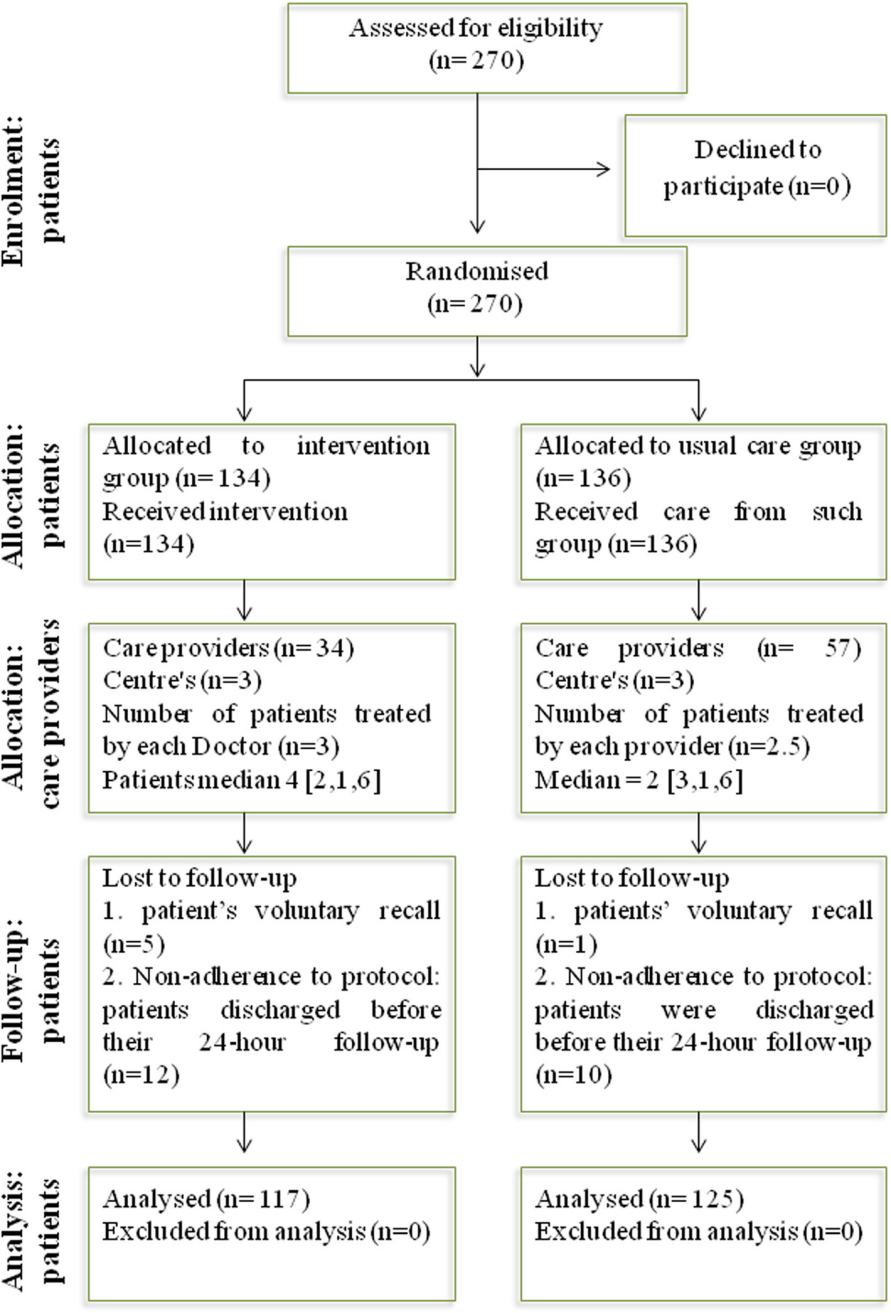
Flow diagram regarding participants

**Fig. 3:**
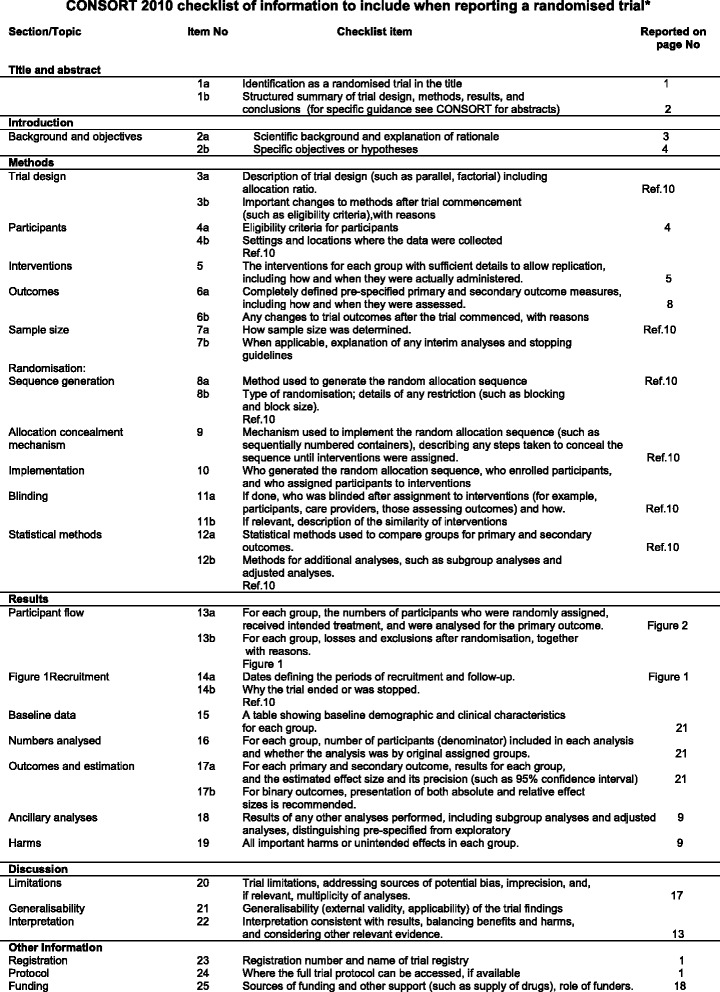
CONSORT checklist of information.

The 270 randomised patients selected by consecutive sampling for the study (134 intervention and 136 controls) were cared for by each of the 3 randomised teams and by 91 admitting doctors. Twenty-eight patients (17 interventions and 11 controls) were excluded; the usual reason for exclusion was they had been assessed and ranked incorrectly during triage, were discharged on the same day or voluntarily decided to leave the hospital and seek care at another hospital. (Figures 2 and 3).

### Patient demographics and clinical characteristics

There were no statistically significant differences between both treatment arms; patients’ had similar characteristics regarding the intervention and standard care arms. The characteristics of patients in the study population are summarised in Table 1.

**Table 1:** The study population’s baseline demographic and clinical characteristics

Characteristics	Total group	Control	Intervention	Value	*p* value
	242 (100)	125 (51.65)	117 (48.35)		
Age, mean ± SD	59 ± 19	58 ± 20	59 ± 18	−0.5355^a^	0.5928
Gender				0.2233^b^	0.6365
Female	140 (57.9)	70 (56.0)	70 (59.8)		
Male	102 (42.1)	55 (44.0)	47 (40.2)		
No. of hospitalisations, mean (IQR, min, max)	0 (1, 0, 12)	0 (1, 0, 10)	0 (1, 0, 12)	−0.2168^a^	0.8285
No. of co-morbidities, mean (IQR, min, max)	1 (2, 0, 4)	1 (2, 0, 4)	1 (1, 0, 4)	0.4219^a^	0.6735
No. of medicines, mean (IQR, min, max)	4 (4, 1, 12)	4 (4, 1, 16)	4 (4, 1, 14)	−0.3299^a^	0.7418
Teaching hospitals				5.1145^b^	0.07752
Fundacion Cardioinfantil	82 (33.9)	36 (28.8)	46 (39.3)		
San Carlos hospital	78 (32.2)	48 (38.4)	30 (25.6)		
Samaritana hospital	82 (33.9)	41 (32.8)	41 (35.1)		

^a^Student’s *t*-test; ^b^Chi-square; SD (standard deviation). IQR, interquartile range; min, minimum; max, maximum

### The effect of the intervention on PADEs

There was very good reliability concerning judging discrepancies’ potential severity. Fleiss’ kappa coefficient was used (κ = 0.829: 0.7-0.96 95 % CI) and consensus was easily achieved in areas of disagreement [14].

The relative risk of at least 1 PADE having occurred per patient was evaluated for each class of PADE; 37 (31 %) of the intervention group had suffered class 3 PADE compared to 70 (56 %) in the control group (0.56 RR: 0.41-0.77 95 % CI). Reduced adjusted relative risk due to the effect of the intervention was 56 %. Regarding class 2 PADEs, 44 (38 %) were identified in the intervention group compared to 93 (74 %) in the control group (0.51 RR: 0.39-0.65 95 % CI); reduced adjusted relative risk due to the effect of the intervention was 50 %. Fifty-three (45 %) class 1 PADEs were detected in the intervention group compared to 80 (64 %) in the control, giving 70 % reduced adjusted relative risk (0.71 RR: 0.56-0.90 95 % CI).

### PADE type and potential severity

The study revealed 811 PADEs (an average of 3.35 per patient; 528 (65 %) occurred on the standard care arm and 283 (35 %) on the intervention arm. Most PADEs were judged to have been capable of causing moderate deterioration (42.6 %), 33.4 % of the PADEs were deemed unlikely to have caused harm and 23.9 % were judged to have been able to cause clinical deterioration. The omission of medication was the most frequently occurring type of PADE able to cause patients’ significant clinical deterioration.

Table 2 shows the types of PADE according to their severity and distribution in intervention and control groups. There was an increase in those related to administration regimen (slow to restart drug therapy or too soon to restart drug therapy) following the intervention and not a reduction, as expected. Slowness to restart drug therapy increased by 6.64 % in class 1, 37.5 % in class 2 and 17.4 % in class 3; an increase in the number of too soon to restart drug therapy type cases was also observed in the intervention group: 0.26 % in class 1, 0.85 % in class 2 and 2.82 % in class 3. Slowness and/or being too early in restarting therapy involving drugs was related to a mismatch between the scheduled administration times at a particular hospital and the patients’ usual administration times. The number of cases involving the intervention group was almost always lower than in the control group regarding the other types of PADE.

**Table 2:** Discrepancy type and potential severity

Type of PADE	Class 1^a^			Class 2^b^			Class 3^c^		
	Total group	Control	Intervention	Total group	Control	Intervention	Total group	Control	Intervention
Incorrect or omitted dose	39 (14.4)	22 (13.2)	17 (16.4)	32 (9.3)	12 (5.0)	20 (18.5)	94 (48.5)	49 (39.8)	45 (63.4)
Therapeutic duplication	4 (1.5)	3 (1.8)	1 (1.0)	0 (0)	0 (0)	0 (0)	1 (0.5)	1 (0.8)	0 (0)
Incorrect or omitted frequency	0 (0)	0 (0)	0 (0)	4 (1.2)	4 (1.7)	0 (0)	2 (1.0)	2 (1.6)	0 (0)
Slow to restart drug therapy	28 (10.3)	13 (7.8)	15 (14.4)	103 (29.8)	43 (18.0)	60 (55.6)	25 (12.9)	8 (6.5)	17 (23.9)
No indication	0 (0)	0 (0)	0 (0)	2 (0.6)	2 (0.8)	0 (0)	0 (0)	0 (0)	0 (0)
Drug omission	190 (70.1)	123 (73.7)	67 (64.4)	187 (54.0)	164 (68.9)	23 (21.3)	70 (36.1)	63 (51.2)	7 (9.9)
Too soon to restart drug therapy	10 (3.7)	6 (3.6)	4 (3.9)	14 (4.1)	9 (3.8)	5 (4.6)	2 (1.0)	0 (0)	2 (2.8)
Inappropriate or omitted route	0 (0)	0 (0)	0 (0)	4 (1.2)	4 (1.7)	0 (0)	0 (0)	0 (0)	0 (0)

^a^Class 1: discrepancies unlikely to cause patient discomfort or clinical deterioration. ^b^Class 2 discrepancies which could cause moderate discomfort or clinical deterioration

^c^Class 3 discrepancies potentially resulting in severe discomfort or clinical deterioration

The association between PADEs and baseline patient characteristics was also evaluated (Table 3). Increased age, being female, the number of comorbidities, the number of hospitalisations and number of drugs being taken were predictors of increased univariate model probability of at least 1 PADE occurring. Regarding the study population’s clinical characteristics in the multivariate model, intervention and ED setting were significant variables regarding a reduced risk of at least 1 PADE occurring per patient. The number of drugs taken at home increased the risk of PADEs occurring.

**Table 3:** Association between patients’ baseline characteristics and at least 1 PADE

Characteristics	Univariate logistic regression		Multivariate logistic regression	
	Odds ratio (95 % CI)	*p*-value	Odds ratio (95 % CI)	*p*-value
Age	1.02 (1.01 - 1.04)	0.0043	1.01 (0.99 - 1.03)	0.2770
Being female	1.76 (1.05 - 2.98)	0.0344	1.56 (0.83 - 2.93)	0.1673
Teaching hospital ED				
San Carlos	0.52 (0.28 - 0.97)	0.0417	0.47 (0.21 - 1.01)	0.0542
La Samaritana	0.28 (0.14 - 0.53)	0.0001	0.22 (0.09 - 0.48)	0.0002
Number of hospitalisations	1.27 (1.03 - 1.63)	0.0369	1.29 (1.00 - 1.74)	0.0713
Number of comorbidities	1.35 (1.07 - 1.73)	0.0137	0.71 (0.49 - 1.00)	0.0511
Number of home medications	1.34 (1.21 - 1.50)	7.75E-08	1.36 (1.19 - 1.58)	1.75E-05
Intervention	0.36 (0.21 - 0.61)	0.0002	0.22 (0.11 - 0.42)	5.64E-06

*CI* confidence interval, *ED* emergency department

Poisson log-linear regression led to obtaining measurements of relative risk associated with each covariate; Table 4 gives the measurements regarding increased or reduced relative risk associated with each co-variable. All variables in the univariate model were seen to be significant (particularly ED setting and intervention) in reducing the risk of a PADE occurring. Age, the number of comorbidities and ED setting associated with the San Carlos hospital were not significant in the multivariate model regarding the possibility of risk occurring.

**Table 4:** Association between patients’ baseline characteristics and the number of class 3 PADEs

Characteristics	Univariate poisson regression		Multivariate poisson regression	
	Estimate (95 % CI)	*p* - value	Estimate (95 % CI)	*p* - value
Age	1.02 (1.01; 1.03)	2.58E-05	1 (0.99; 1.01)	0.4377
Being female	2.03 (1.82; 2.83)	1.19E-05	1.49 (1.06; 2.12)	0.0235
Teaching hospital ED				
San Carlos	0.73 (0.52; 0.99)	0.0462	0.99 (0.7; 1.4)	0.9731
La Samaritana	0.4 (0.27; 0.58)	2.12E-06	0.46 (0.3; 0.69)	0.0003
Number of comorbidities	1.32 (1.17; 1.49)	3.05E-06	0.91 (0.78; 1.06)	0.2275
Number of hospitalisations	1.16 (1.08; 1.22)	4.66E-06	1.13 (1.04; 1.22)	0.0019
Number of home medications	1.23 (1.19; 1.3)	<2e-16	1.19 (1.13; 1.26)	1.18E-09
Intervention	0.62 (0.46; 0.83)	0.0012	0.6 (0.44; 0.8)	0.0007

*CI* confidence interval, *ED* Emergency Department

## Discussion

The intervention was associated with a significant reduction in the severity of any type of PADE concerning admission to an ED, shown by the relative risk of at least 1 PADE occurring in each class. Our results were consistent with previous studies [8, 15]. Schnipper *et al*., [7] found that the effectiveness of having a pharmacist involved in healthcare acquiring patients’ medication histories led to reducing the occurrence of at least 1 PADE per patient; such result was very similar to that found in this study. Regarding PADE potential severity, there were fewer medication omissions in the intervention, probably due to doctors having more information available when prescribing medication during ED consultation and as such information could have been verified together with patients.

The study also detected an unexpected rise in the amount of PADEs related to restarting therapy (promptness or slowness) in the intervention group. Percentage variation regarding slowness was greater for both groups and all types of PADE severity. Greater promptness in administering medicine could have been associated with doctors giving priority to critical events in an ED and patients’ home administration regimens being omitted. Such was the researchers’ perception as it was not a previously established result and requires further investigation. The potential risks of unsuitable management regarding administration frequency could have been associated with the probable appearance of therapeutic failure due to drug concentration in blood not reaching the therapeutic minimum, e.g. when delay in administering medication was more than 24 h or, contrarily, administering medicines with greater frequency than that established in posology could have eventually provoked an unexpected increase in drug concentration in blood, thereby increasing the risk of a PADE occurring. All of the foregoing should be evaluated by a doctor and would depend on the type of medicament being taken and a patient’s clinical condition.

An increase in PADEs may be explained by many events (according to the researchers’ un-programmed observations), but may have been caused because the hospitals had previously established nursing services’ medicament administration times/schedules as their current hospital policy. A pharmacist-acquired MH in an ED may have guaranteed that doctors had more information available regarding patient medication during consultation, thereby reducing prescription errors and contributing towards more widespread introduction of new medication regimes [16].

Analysing the explanatory models’ results revealed that the PADEs reported in this study agreed with findings from other studies. Gender was associated as a characteristic predicting an increased risk of a PADE happening in the present study (Tables 3 and 4). Other studies have concluded that differences regarding pharmacokinetics, pharmacodynamics and medication side-effects were gender-dependent and may have reflected response profiles concerning drugs’ different effects. The effect of gender on drug response represents a very recent field of research for most drugs; the effect of specific dosage and administration route have begun to be explored even more recently, thereby indicating the need for specific gender analysis as the only suitable procedure for detecting such differences (Mei *et al*., [17], Anderson *et al*., [18]).

The high number of co morbidities has increased the number of hospitalisations due to the association between variables and the number of medicines being taken. The multivariate model showed that only the number of medicaments and an increased risk of class 3 PADE occurring were statistically significant. The intervention was associated with a significant reduction in PADEs at the Samaritana hospital but not at the other hospitals involved in the study (Table 4).

It was thus noted that some differences concerned particular hospitals in the study, particularly the Samaritana hospital, probably due to specific features concerning the service offered during ED admission and maybe due to the patient:doctor ratio being the lowest of the three hospitals and more time being spent on average during consultation. The aforementioned points are speculative since the patient:doctor ratio and consultation duration were not variables which were measured at the start of the study and thus do not represent a conclusion resulting from an analysis of the information made available during the study. Although the hospitals participating in the study were selected as they had very similar general characteristics, it was revealed that an ED healthcare setting significantly affected the risk of PADEs occurring.

Both models led to concluding that the intervention reduced adverse events occurring due to patients’ medication errors and that percentage reduction was statistically significant, probably due to the intervention group having a more complete MH available and MedRec making this safer.

The models were consistent regarding the clinical variables supporting their explanation whilst differences between estimated models showed that sociodemographic variables (age and gender) were significant in the Poisson model. This could have been due to a strong association of such factors with the number of PADEs and not with the presence of at least 1 PADE. Identifying a single risk can be considered as one of four important steps regarding the safe use of medicines: risk detection, risk assessment, risk minimisation and risk communication. However, a typical individual medicinal product will have multiple risks attached to it and individual risks will vary in terms of severity, a particular patient and public health impact. The combination of information regarding potential adverse drugs events could thus ensure that the benefits exceed the risks by the greatest possible margin both for individual patients and the population as a whole.

This study had several limitations. Despite the study having been conducted in teaching hospitals, it may not be possible to extrapolate the results to other settings because an ED setting was a factor regarding the risk of a PADE occurring. Future research could examine the effect of an ED admission setting and blocking; a cluster study should thus be carried out.

Error rates may differ regarding services other than an ED concerning admissions which are elective or involve a transfer from another healthcare facility, or concern patients taking more than 1 medication. Our findings may not have been representative of other institutions which do not use MedRec on admission. Eligible patients were not followed-up beyond the study; the effect of such ED admission process on medical outcome is thus unknown.

The rating method used for assessing the potential severity of discrepancies and the admission medication order form (F1) questionnaire used during a MH interview have not been validated. Intra-researcher agreement was not evaluated as interviewing the same patient twice could have led to recall bias.

The hospitals involved in this study are currently developing a MedRec pathway which will incorporate some strategies based on the findings from the present study. The next phase of this study will involve an assessment of medication discrepancies once the new MedRec protocol is in place.

The potential risk of adverse events was evaluated by groups of drugs (e.g. cardiovascular or gastrointestinal drugs) and not by specific medicaments (e.g. digoxin or warfarin). This was due to the large amount of drugs being taken by the patients in the study. Some risks producing moderate clinical deterioration may not have been considered, because only those threatening a patient’s life were taken into account (i.e. class 3), thereby limiting the analysis.

## Conclusions

It was concluded that potentially adverse drug events occur for many patients during the transition of care from home to hospital. Patient safety-focused MedRec during admission to an ED involving a pharmacist and drawing up a complete MH could thus contribute towards reducing the risk of PADEs occurring and could improve patients’ medicament-based follow-up therapy.
